# Improved artificial bee colony algorithm for vehicle routing problem with time windows

**DOI:** 10.1371/journal.pone.0181275

**Published:** 2017-09-29

**Authors:** Baozhen Yao, Qianqian Yan, Mengjie Zhang, Yunong Yang

**Affiliations:** 1 Automotive Engineering College, Dalian University of Technology, Dalian, P.R., China; 2 Transportation Management College, Dalian Maritime University, Dalian, P.R., China; Beihang University, CHINA

## Abstract

This paper investigates a well-known complex combinatorial problem known as the vehicle routing problem with time windows (VRPTW). Unlike the standard vehicle routing problem, each customer in the VRPTW is served within a given time constraint. This paper solves the VRPTW using an improved artificial bee colony (IABC) algorithm. The performance of this algorithm is improved by a local optimization based on a crossover operation and a scanning strategy. Finally, the effectiveness of the IABC is evaluated on some well-known benchmarks. The results demonstrate the power of IABC algorithm in solving the VRPTW.

## Introduction

The Vehicle Routing Problem (VRP) was first described by Dantzig and Ramser in 1959 [1]. The problem involves determining the delivery routes, which start and end at the depot and also serve all the customers. Each customer is visited once and the total demand of all customers in a specific route can’t exceed the capability of the vehicle. The goal of the VRP is to minimize the total cost, where cost can be defined as distance or time [2]. In 1981, Lenstra and Rinnooy Kan [3] proved that the VRP is NP-hard. Its main drawback is the well-known curse of dimensionality. Constraint programming is considered as an efficient method for giving the optimal solution [4] but the time to find the optimal is prohibitive in large problems. Some studies improved constraint programming to solve the VRP: Backer et al. [5] developed a method using iterative improvement techniques within a Constraint Programming framework to get a good solution under a relatively short computing time; Ozfirat and Ozkarahan [6] introduced an algorithm which decomposing the problem into smaller scale ones firstly and then solve it by constraint programming; Guimarans et al. [7] proposed a method of combining Constraint Programming, Probabilistic Algorithms and Lagrangian Relaxation.

The vehicle routing problem with time windows (VRPTW) is a variant of the VRP that has an additional time window constraint. In the VRPTW, a fleet of vehicles set off from a depot to serve a number of customers, which have various demands and specific time windows. The objective of the VRPTW includes maximizing the sum of the on-time delivery probabilities to customers, minimizing the expected total cost, and some others [8]. The VRPTW, as an extension of the VRP, has also been proved to be an NP-hard problem [9]. The time window require the time constraint and the traffic speed prediction is very important in the delivery routes in VRPTW [10–11]. In addition, Heuristic algorithms were considered effective methods to solve this combinational problem [12–16]. Thangiah *et al*. [9] and Wainwright [14] developed genetic algorithms to solve the VRPTW. The former's objective was to minimize the distance of vehicle routes, while the latter study had two objectives: use the least vehicles, and minimize the total costs under the condition of the first objective. Zhang et al. [17] improved tabu search heuristic algorithm by incorporating a route reduction mechanism to reduce the number of required vehicles. Yao et al. [18] proposed an improved particle swarm optimization to solve carton heterogeneous vehicle routing problem with a collection depot. The results suggested that the proposed algorithm was an effective approach for the problem.

When a bee colony looks for nectar, they often divide into three initial groups: leaders, scouts and onlookers. By communicating information among the three roles, the colony gathers nectar quickly and efficiently. The Artificial Bee Colony (ABC) algorithm is inspired by the behavior of bees as they search for nectar and some studies have proposed improvements to the algorithm. Seely [19] proposed a self-organization simulation model for the colony, where the entire colony collaborates to complete a complicated problem, such as building the hive or harvesting pollen. Karaboga [20] successfully applied the bee colony algorithm to the numerical optimization of functions and proposed a systematic artificial bee colony algorithm. In 2007, ABC theory was further applied to solve restrictive numerical optimization problems by Basturk and Karaboga [21], and the authors presented promising results.

As a novel heuristic algorithm, the ABC algorithm has been successful in solving complex combinational optimization problems. Özbakir *et al*. [22] presented an ABC algorithm to solve generalized assignment problems with an ejection chain neighborhood mechanism. Koudil *et al*. [23] attempted to use an ABC algorithm to solve an integrated partitioning/scheduling problem in codesign. Karaboga *et al*. [24] proposed a modified ABC algorithm to determine the parameters of a Schottky barrier diode. Cuevas *et al*. [25] developed an ABC algorithm to solve an image segmentation problem by computing threshold selection. Zhang *et al*. [26] addressed problems using three enhanced versions of the original ABC algorithm, based on multi-species co-evolution. Huo *et al*. [27] proposed a Discrete Gbest-guided ABC algorithm for cloud service composition. AlMuhaideb *et al*. [28] presented a two-phase strategy that combined ant colony optimization and the ABC algorithm. Other researches involving ABC algorithms can be found in the literatures [20, 29]. These successful applications motivated us to apply the ABC in this paper to generate and optimize a variety of potential routes to solve the VRPTW problem.

The ABC algorithm is a popular optimization algorithm, but it is often easily trapped in local optima before it finds the global optimum. Besides, if the current search settles on obviously bad information and inputs that information into the next search stage, search accuracy will decrease. Thus, it is necessary to expand the solution space of the algorithm or, equivalently, to expand the diversity of its search information. Crossover operation of the genetic algorithm is an effective way to expand the range of the solution search. Vaira et al. [30] investigated the crossover operators for a vehicle routing problem and proved its superiority. Other researches using crossovers to expand solution space include Jih et al. [31], Misevičius et al. [32]; Kumar et al. [33]. Scanning strategy judges the relationship between two paths from geometric knowledge and makes adjustments if the new solution satisfies the requested intersection and time window constraints. It can prevent bad information—e.g. unnecessary intersections between two paths—from entering the following search iteration. The main contribution of this paper is combining the ABC algorithm with crossover and scanning strategy, which accelerate the convergence speed and improve the solution to VRPs compared with conventional ABC algorithms.

The remainder of this paper is organized as follows. In Section 2, we construct a mathematical model for the VRPTW. The ABC algorithm and the proposed strategies outlined above are detailed in Section 3. Section 4 discusses the computational results and Section 5 concludes the paper. Finally, a list of the notation used in the proposed algorithm is presented in the S1 Table.

## Problem description

A typical VRPTW specifies: the service time constraint, demand of each customer and the capacity of each vehicle. The routes must be designed so that each point is visited once by one vehicle and all routes start and end at the depot. The total demand of all points on one particular route cannot exceed the capacity of the vehicle. The service time of each customer must be in the time constraints of the customers.

The VRPTW model in this paper is represented as a weighted graph, *G* = (*V*, *E*), where *V* = {1,2,3,⋯,*N*} represents the vertex set and $\underline{N}$ is the number of nodes in the graph, $\underline{E}$ represents the edge set; *c*_*ij*_(*c*_*ij*_ > 0, *c*_*ii*_ = ∞, *i*, *j* ∈ *V*) represents the distance between a pair of vertices. The notation used in this model are listed as follows in Table 1.

**Table 1:** Notation of the model.

*V*	Set of depots and customers
*K*	Set of vehicles
*Q*	The capacity of each vehicle
*q*_*i*_	The demand of each customer, *i* ∈ *V*
*s*_*ei*_	The start time of serving customer, *i* ∈ *V*
*s*_*li*_	The end time of serving customer, *i* ∈ *V*
*s*_*i*_	The time needed to complete the mission of customer, *s*_*i*_ = *s*_*li*_ − *s*_*ei*_, *i* ∈ *V*
*t*_*ij*_	The travel time between customer *i* and *j*, *i*, *j* ∈ *V*
*t*_*i*_	The arrival time at customer *i*, *i* ∈ *V*
*w*_*i*_	The wait time at customer *i*, *i* ∈ *V*
*e*_*i*_	The earliest arrival time at customer *i*, *i* ∈ *V*
*l*_*i*_	The latest arrival time at customer *i*, *i* ∈ *V*
*i* = 0	The start node or the depot of VRP
*T*_*k*_	The maximum route time allowed for vehicle *k*, *k* ∈ *K*
Decision variables	
*x*_*ijk*_	*x*_*ijk*_ = 1, if the edge from customer *i* to *j* is visited by vehicle *k*; *x*_*ijk =*_ 0, otherwise. *i*, *j* ∈ *V*; *k* ∈ *K*
*P*	The maximum number of vehicles

For the sake of convenience, several assumptions are made in this paper: (1) Customer demands can be split and the maximum possible demand of each customer is smaller than the vehicle capacity; (2) a customer’s actual demand is only re-vealed when the vehicle arrives at that customer’s location and the customer’s earliest acceptable service time (i.e. the lower bound of the time window) begins; (3) service times at customer locations are neglected for clarity. (4) We assume *t*_*ij*_ = *c*_*ij*_.

The model is described as follows:

$$\mathrm{Min}\sum_{i=0}^{N}\sum_{j=0j\neq i}^{N}\sum_{k=1}^{P}c_{ij}x_{ijk}$$ (1)

$$\sum_{k=1}^{P}\sum_{j=1}^{N}x_{ijk}\leq P\;i\in 0$$ (2)

$$\sum_{j=1}^{N}x_{ijk}=\sum_{j=1}^{N}x_{jik}\leq 1\;i\in \{0\}\;k\in \{1,\ldots ,P\}$$ (3)

$$\sum_{k=1}^{P}\sum_{j=0,j\neq i}^{N}x_{ijk}=1\;\mathrm{for}\;i\in \{1,\ldots ,N\}$$ (4)

$$\sum_{k=1}^{P}\sum_{i=0,i\neq j}^{N}x_{ijk}=1\;\mathrm{for}\;j\in \{1,\ldots ,N\}$$ (5)

$$\sum_{i=1}^{N}q_{i}\sum_{j=0,j\neq i}^{N}x_{ijk}\leq Q\;\mathrm{for}\;k\in \{1,\ldots ,P\}$$ (6)

$$\sum_{i=1}^{N}\sum_{j=0,j\neq i}^{N}x_{ijk}(t_{ij}+s_{i}+w_{i})\leq T_{k}\;\mathrm{for}\;k\in \{1,\ldots ,P\}$$ (7)

$$t_{0}=w_{0}=s_{0}=0$$ (8)

$$x_{ijk}(t_{i}+t_{ij}+s_{i}+w_{i})\leq t_{j}\;\mathrm{for}j\in \{1,\ldots ,N\}$$ (9)

$$e_{i}\leq (t_{i}+w_{i})\leq l_{i}\;\mathrm{for}\;i\in \{1,\ldots ,N\}$$ (10)

The objective is to design a network that can minimize the total travel length under the conditions of all constraints. The constraints are defined as follows:

(2) The maximum number of routes constraint: to specify the maximum *P* routes going out of the depot.
(3) Travel constraint: ensures every route starts and ends at the central depot.
(4)–(5) Service constraints: to assume that every customer node can be visited exactly once by one vehicle.
(6) The capacity constraint: to assume the quantity of a vehicle cannot exceed its capacity.
(7) The maximum travel time constraint: to limit the travel time to less than the maximum.
(8)–(10) The time windows constraints: to define the time windows.

As the ABC algorithm is easily trapped in local optima, we present an improved artificial bee colony (IABC) algorithm to avoid this limitation in our investigation of the vehicle routing problem.

## Improved artificial bee colony algorithm

Artificial bee colony (ABC) algorithm is a relatively new optimization method, which simulates the acts of the real bees looking for nectar in nature. It has been successfully applied to solve a series of complex combinational optimization problems. In order to overcome some limitations of the standard ABC algorithm, an improved ABC algorithm is presented in this paper. And in this section, we firstly introduce the fundamental rules of ABC algorithm, then two local optimization methods that prevent the algorithm trapped in local optimal are presented.

### The fundamental rules of ABC

**Fig 1:**
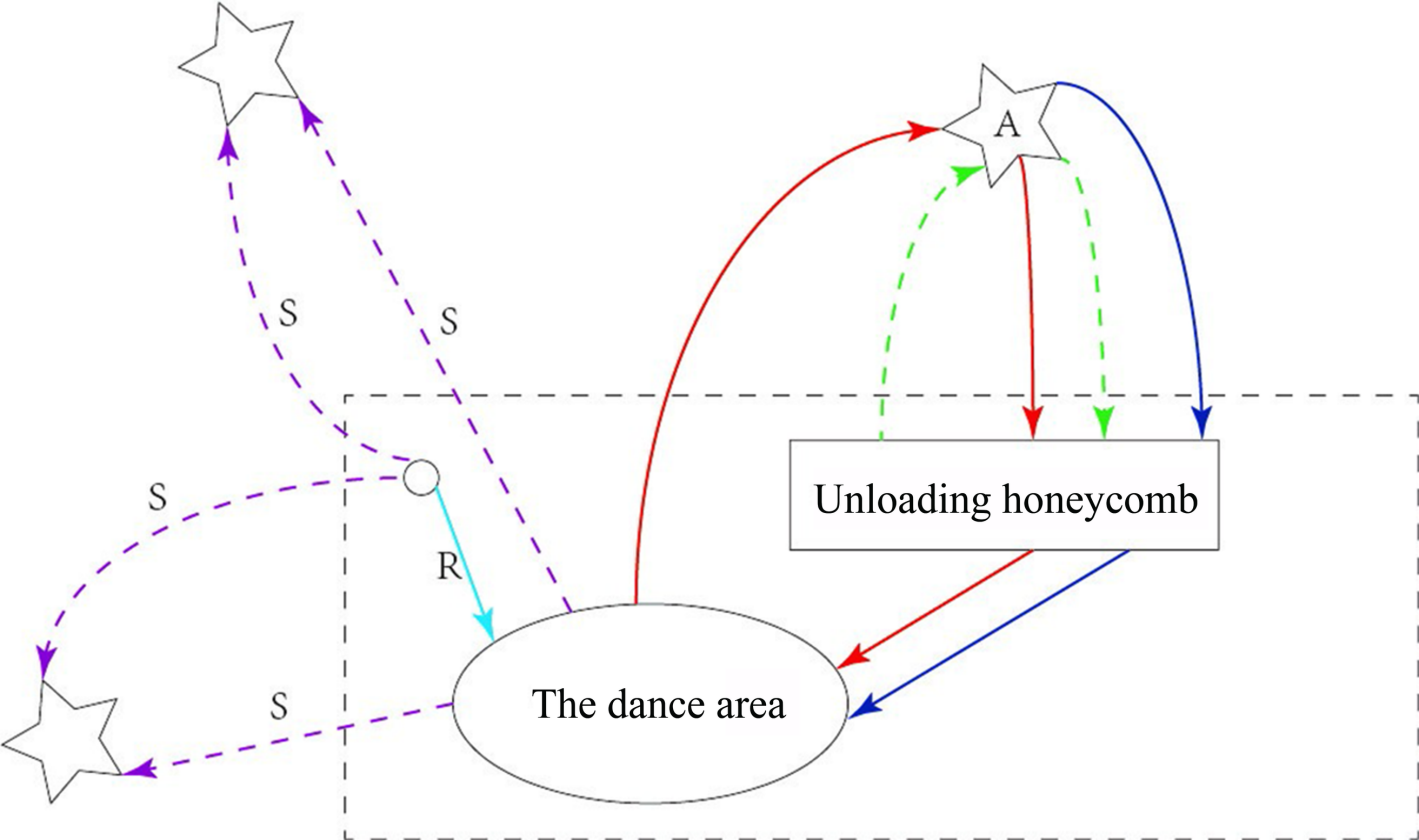
Behavior of honey bee foraging for nectar.

An ABC consists of three main populations: employed bees (or called leaders), onlookers and scouts. Employed bees are responsible for the mining process and gathering required information. Scouts undertake the exploitation of new food sources. Whereas onlookers are in charge of sharing information with employed bees and scouts by communicating in the dance area. The three roles are interchangeable among the individuals. For example, in Fig 1, a bee initially has two choices; it can scout for food sources (role S in Fig 1), or follow the leader as an onlooker after witnessing the leader’s dance (role R in Fig 1). When a scout has found the food source and begins collecting nectar, it becomes a leader. This bee then returns to the honeycomb and unloads the food. At this time, it has three choices; abandon the food source and become a scout or onlooker (blue line in Fig 1), continue the leader role and dance to recruit more bees for food sourcing (red line in Fig 1), or collect nectar from the food source without recruiting other bees (green line in Fig 1) (Liu *et al*. [34]).

In the ABC algorithm, each alternative solution is called a nectar source. The nectar collecting process is similar to the process of searching for the optimum solution. The quality of each nectar source is determined by its corresponding fitness value. The number of solutions (*S*) equals the number of leaders or onlookers. The position of food source *i* is represented by a *D*-dimensional coordinate vector *u*_*i*_(*u*_*i*1_,*u*_*i*2_,⋯,*u*_*id*_,⋯,*u*_*iD*_)^*T*^ ∈ {*u*_*i*_|1 ≤ *i* ≤ *S*,*i* is integer}. So the ABC algorithm first obtains an original population with *S* solutions, where each solution *u*_*i*_(*i* = 1,2,⋯,*S*) is a *D*-dimensional vector. Then during each iteration, each leader finds a new food source by conducting a cyclic neighborhood search on its originally old food source. The cycle number is denoted by *C*(*whereC* = 1,2,⋯,*M*). After the new food position is generated, its corresponding fitness value need to be evaluated.

If the nectar found at the present food source (solution) is of higher quality (fitness) than the former nectar, the old food source is replaced by the new source; otherwise, the position of the old food source is unchanged. As the search approaches the best solution, the neighborhood range becomes smaller. Once all leaders have completed their search work, they return to the dance area and deliver the nectar information of the food source to onlookers. Then, each onlooker selected a food source according to the roulette wheel selection method. The higher the nectar content of a food source is, the higher the probability that that food source will be chosen by onlookers.

Having chosen a food source, onlookers either select a neighborhood search around the food source, or keep the current solution. Furthermore, the ABC algorithm imposes a parameter limit on the number of updates. If a solution is not improved after consecutive cycles, it has sunk into a local optimum and should be abandoned. The corresponding leader then converts into a scout and searches for new food source randomly. After the scout finds a new food source, the scout becomes a leader again. After each leader is assigned to a food source, another iteration of the ABC algorithm begins. The whole process is repeated until a stopping condition is met. For more details about the ABC algorithm, interested readers should refer to Karaboga [20,35] and Szeto [29].

Based on the preceding analysis, four selection processes are specified in the ABC algorithm; first is the global selection process, in which onlookers seek a good food source; second is the local selection process, by which leaders and onlookers search the neighborhood; third is a greedy selection process in which all artificial bees judge and compare the old and new food sources, then retrieve the best solution; and fourth is a stochastic selection process that identifies a food source.

In the application of vehicle routing problem, the bees construct paths in different ways depending on their roles. Leaders only retrace the last path of the iterative procedure without changing the situation. The scouts and followers choose the next nectar source as follows:

$$p_{ij}^{k}(t)=\{\begin{array}{cc} \frac{{\left[S_{ij}(t)\right]}^{\alpha }{\left[f_{y}(t)\right]}^{\beta }}{\sum_{Allowd_{i}^{k}}{\left[S_{ij}(t)\right]}^{\alpha }{\left[f_{y}(t)\right]}^{\beta }} & j\in A_{i}^{k}\\ 0 & otherwise \end{array}$$ (11)

where $p_{ij}^{k}(t)$ represents the probability that bee *k* travels from node *i* to *j* in iteration *t*. $A_{i}^{k}=\Phi -Tabu_{k}$ denotes the available nodes for bee *k* (*i*.*e*., the points that satisfy all constraints but have not been previously visited by the bee). Tabu *k* is the tabu list of bee *k*, which prevents the nodes already visited by bee *k* from being repeatedly visited. In some applications (*i*.*e*., router optimization), it is also convenient that the bee can return through the original path. *s*_*ij*_(*t*) and *f*_*ij*_(*t*) represent the bee colony and heuristic information, respectively, while *α* and *β* are the weight coefficients.

The control information differs between scouts and followers. For a scout, the control information is given by

$$s_{ij}(t)=\frac{1}{l}$$ (12)

where *l* is the number of unvisited nodes.

For the follower, the information is given by

$$s_{ij}(t)=\{\begin{array}{ll} \{\begin{array}{ll} n & \;\mathrm{Select}\;\mathrm{the}\;\mathrm{path}\;\mathrm{of}\;\mathrm{scout}\\ \frac{1-r}{l-1} & \mathrm{Abandon}\;\mathrm{the}\;\mathrm{path}\;\mathrm{of}\;\mathrm{scout} \end{array} & \mathrm{Optional}\;\mathrm{paths}\;\mathrm{contain}\;\mathrm{the}\;\mathrm{scout}\;\mathrm{path}\\ \frac{1}{l} & \mathrm{Abandon}\;\mathrm{the}\;\mathrm{path}\;\mathrm{of}\;\mathrm{scout} \end{array}$$ (13)

where *r* represents the guiding strength of the leader, and *l* represents the number of unvisited nodes, as before.

However, the above ABC easily becomes trapped in local optima. Moreover, a local optimum is difficult to escape because of the limited diversity of solutions, and the current solution may contain bad information that is passed to the next solution. To overcome these problems, we introduce the crossover operation and a scanning strategy for local optimization.

### Local optimization

Due to the weaknesses of the standard ABC, it is necessary to expand the solution space and prevent bad information entering the following search. In this section, crossover operation is first introduced to expand the range of the solution search, then, scanning strategy is developed to eliminate the effect of bad information.

#### Crossover operation

**Fig 2:**
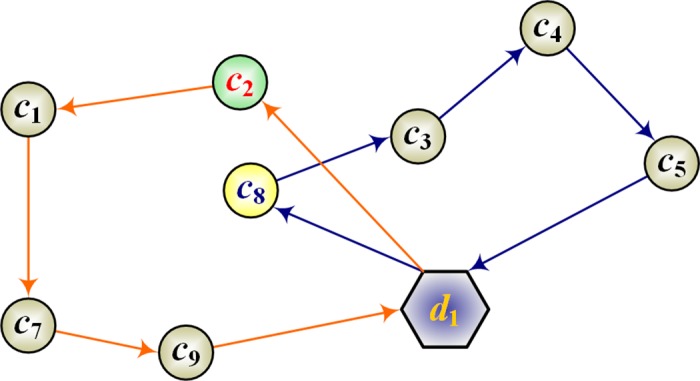
Point selection in the crossover operations (clients *c*_2_ and *c*_8_ are selected).

**Fig 3:**
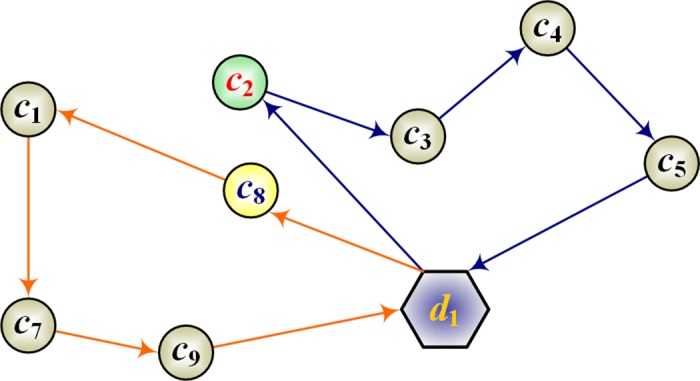
Point exchange in the crossover operation.

In local optimization, the crossover operation effectively prevents the algorithm from trapping in a local optimum and reaches further solutions in the search space [36]. In this paper, the performance of ABC is improved by a simple crossover operation, which proceeds as follows:

**Step 1**. Randomly select two paths from the solutions. Among the points in each path, randomly select one crossover point. For example, suppose that clients *c*_8_ and *c*_2_ are selected, as shown in Fig 2. And by exchanging *c*_8_ and *c*_2_, we obtain a new solution (Fig 3).

**Step 2**. Because the clients are selected randomly, the solution is likely to be inferior. Therefore, the local optimization of the solution must be improved by a local search algorithm. In this paper, the solution is improved by a 2-opt algorithm (Yu *et al*., 2012).

Similar to the genetic algorithm, we introduce a crossover rate (*p*_*m*_) that decides whether a solution needs a crossover. If *p*_*m*_ is too large, it will slow the convergence speed of the algorithm. In the early stages, the optimization should usually be performed over the largest possible search space.

Over time, the accuracy of the optimization will gradually improve. A large jump in accuracy will impede the convergence. Therefore, we adopt an adaptive algorithm that computes the crossover rate as follows:

$$p_{m}(t)=p_{m}^{min}+{\left(p_{m}^{max}-p_{m}^{min}\right)}^{1-t/T}$$ (14)

where $p_{m}^{min}$ and $p_{m}^{max}$ represent the minimum and maximum crossover rates, respectively,

$p_{m}^{min}=1/H$ (*H* is the number of clients in the net),
$p_{m}^{max}=1/N$ (*N* is the number of distribution centers),

and *T* and *t* denote the maximum and current iteration generations, respectively.

#### Scanning strategy

**Fig 4:**
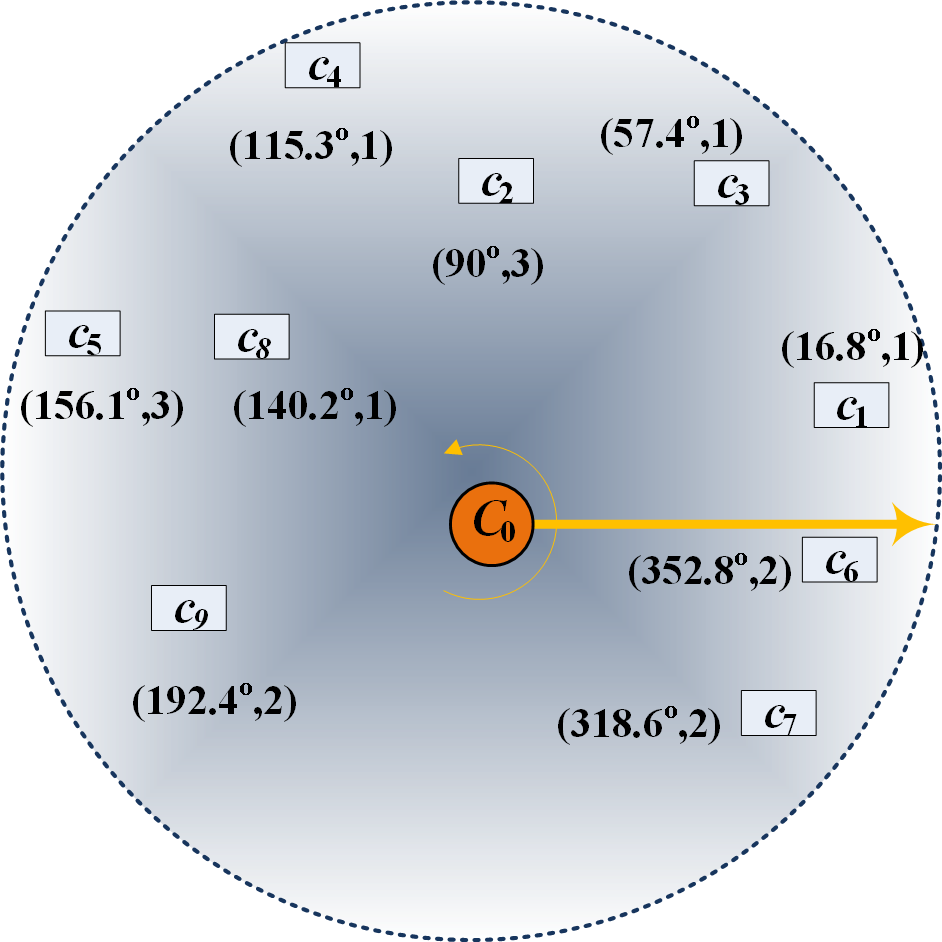
Distribution of customers around the central depot (*c*_0_) in the coordinate system of the scanning strategy.

If two paths in an optimization program intersect at one point, the local optimization can be effectively performed by eliminating that crossover point [37, 38]. Most researches find the crossover point by an inefficient enumeration method (that is, by comparing all paths). Especially in large-scale VRPs, this algorithm requires a long computing time. In this paper, the local optimization program also reduces the crossover phenomenon, but quickly finds the crossover phenomenon by a scanning method [39]. This rapid technique greatly improves the efficiency of the local optimization algorithm. The scanning method proceeds by the following steps:

**Step 1** Initialization. The proposed scanning strategy considers the angle between the customer point and central depot; therefore, the locations of these points must be known. To this end, the scanning strategy establishes a coordinate system with the origin set to the central depot coordinates, then draws a line from the origin to the customer point in two-dimensional space. As an example, Fig 4 shows the spatial distribution of customer points around the central depot. Each customer is initialized to note its location and corresponding paths. For example, if customer *c*_4_ is located 115.3° from the horizontal and the selected path is 1, the (location, path) of that customer is initialized to (115.3^o^, 1).

**Fig 5:**
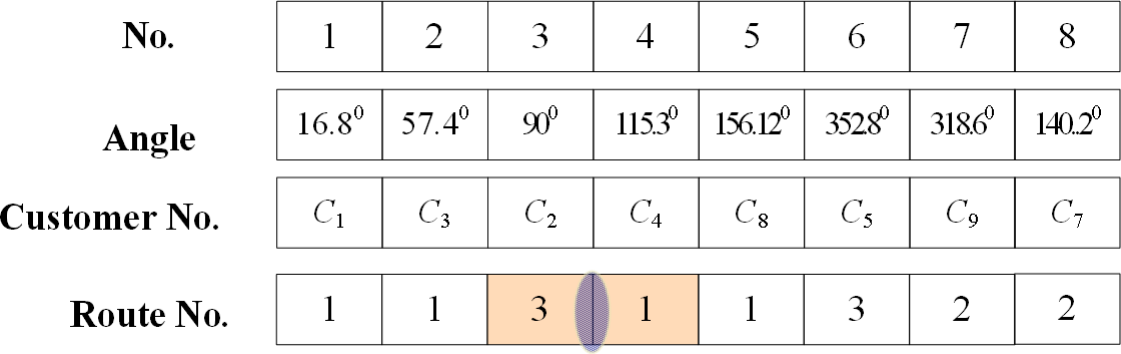
Customer sorting by angles.

**Step 2** Search for the potential crossover paths based on the scanning strategy. The customer points are sorted by their angular values, and their path numbers are recorded as shown in Fig 5. Starting from the customer with the smallest angle, all paths containing the same customer are scanned. The path number can be changed (e.g., from customer *c*_3_ to *c*_2_, the path number changes from 1 to 3). However, the new path may have been already scanned (e.g., from customer *c*_2_ to *c*_4_, the path number changes from 3 to 1), a phenomenon called fallback. If the customer points in one path are continuous and never encounter the fallback phenomenon, that path does not cross any other path (i.e., path 2 in the present example). The fallback phenomenon indicates a possible intersection. The possibly intersecting path is then checked in the tabu list. If the path resides in the tabu list, the algorithm determines whether there is a crossover point between two paths (Step 3). If the result is negative, the scanning continues. If the scan reaches the final customer point without encountering the fallback phenomenon, the algorithm advances to Step 6.

**Step 3** Compare all sections between two paths to judge whether they intersect. The judgement of an intersection between two paths is as follows:

Assume that there exist two paths *ab* and *cd*, in which path *ab* has endpoint coordinates (*x*_1_, *y*_1_) and (*x*_2_, *y*_2_) and path *cd* has endpoint coordinates (*v*_1_, *w*_1_) and (*v*_2_, *w*_2_).

1) If *x*_1_ ≠ *x*_2_ and *v*_1_ ≠ *v*_2_

First, the slopes *θ*_1_ and *θ*_2_ of paths *ab* and *cd* are respectively computed as follows:

$$\theta_{1}=(y_{2}-y_{1})/(x_{2}-x_{1})$$ (15)

$$\theta_{2}=(w_{2}-w_{1})/(v_{2}-v_{1})$$ (16)

**Fig 6:**
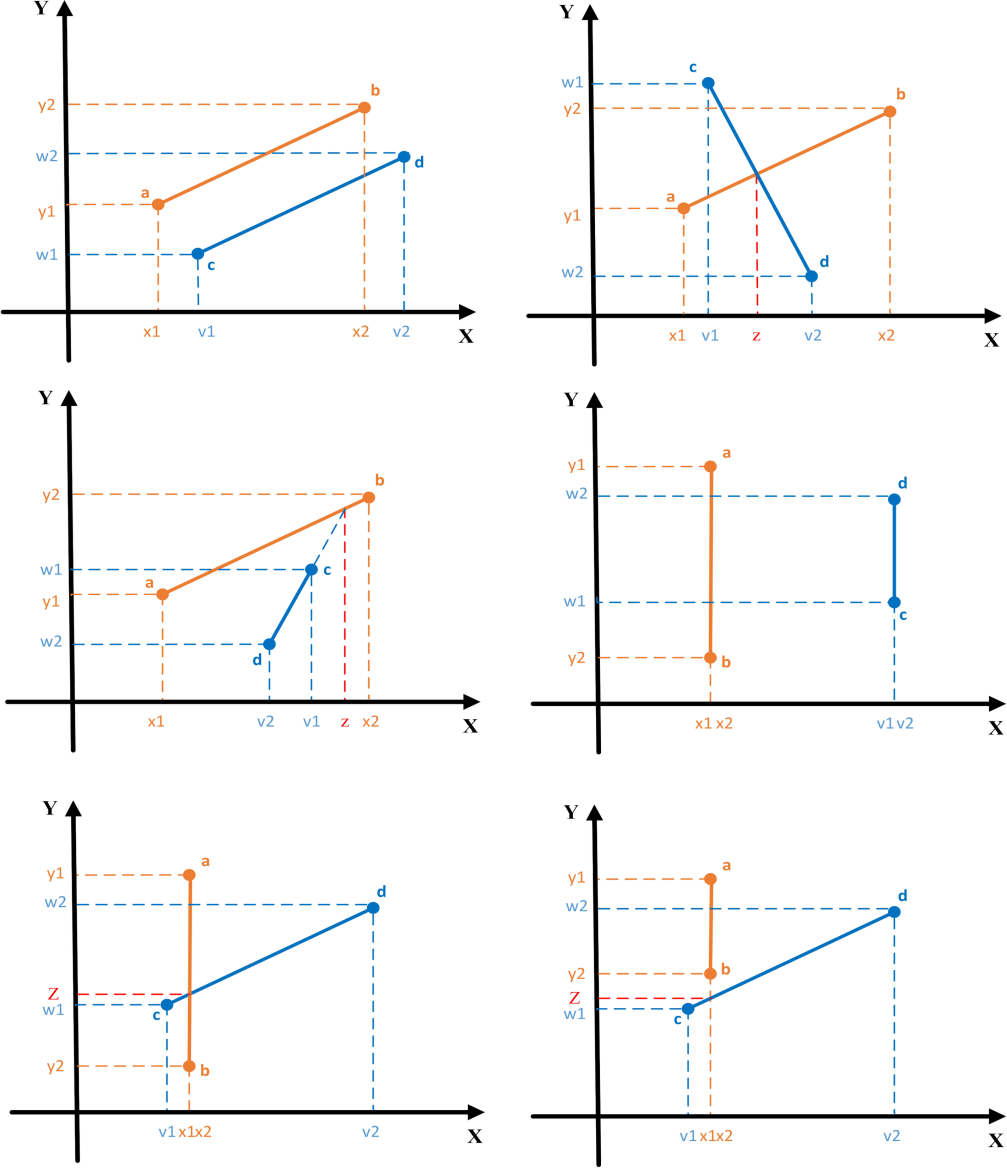
Six situations of two paths.

If *θ*_1 =_ *θ*_2_, the two paths cannot intersect (Fig 6 (A)); otherwise, they may or may not intersect. In the second case, the existence of the intersection is checked by calculating the coordinates of the potential intersection. The abscissa of the potential intersection is:

$$z=(b_{2}-b_{1})/(\theta_{2}-\theta_{1})$$ (17)

$$b_{1}=\left(x_{1}\times y_{2}-x_{2}\times y_{1}\right)/\left(x_{1}-x_{2}\right)$$ (18)

$$b_{2}=\left(v_{1}\times w_{2}-v_{2}\times w_{1}\right)/\left(v_{1}-v_{2}\right)$$ (19)

where, *b*_1_ and *b*_2_ are two judgment parameters.

If *z* lies between *x*_1_, *x*_2_, and *v*_1_, *v*_2_, then the intersection exists (Fig 6 (B)); otherwise, the intersection is absent (Fig 6(C)).

2) If *x*_1_ = *x*_2_ or *v*_1_ = *v*_2_

If *x*_1_ = *x*_2_ and *v*_1_ = *v*_2_, the two paths cannot intersect (Fig 6(D)).

If *x*_1_ = *x*_2_ and *v*_1_ ≠ *v*_2_, the vertical axis of the potential intersection *Z* is computed by inserting *z* = *x*_1_ into the linear equation of path *cd*. If *Z* lies between *y*_1_ and *y*_2_, the two paths intersect (Fig 6(E)); otherwise, they do not intersect (Fig 6(F)). The case *x*_1_ ≠ *x*_2_ and *v*_1_ ≠ *v*_2_ is treated similarly.

If the two paths intersect, the scanning strategy proceeds to Step 4; otherwise, the traversal continues. If no intersection has been found after the traversal, the strategy proceeds to Step 5, which adds these two paths to the tabu list to avoid repeated judgements in subsequent iterations.

**Step 4** The generation of new tours is ensured by the 2-opt algorithm. If the two new tours can improve the solution, they replace the old tours; otherwise, the strategy returns to Step 2.

**Step 5** Judgments are terminated if no feasible crossover is found or if the algorithm has reached the specified iteration limit; otherwise, the strategy returns to Step 2.

## Case studies

### Determination of scouter and leader proportions

The proportions of scouters and leaders in the colony are denoted as *PScouter* and *Pleade*r respectively. These proportions largely determine the performance of the ABC algorithm. In particular, *PScouter* reflects the strength of the randomness in the path searching; the larger the *PScouter*, the less likely that the colony will select a previously visited path. In this way, the colony can extend the solution space. However, the strengthened search randomness tends to reduce the convergence speed. Meanwhile, *Pleader* reflects the strength of the convergence factor in the path searching; the larger the *Pleader*, the more likely that the colony will select the best previously visited path. Thus, a large *Pleader* improves the capability of maintaining the current optimal solution, but increases the chance of falling into a local optimum. In summary, *Pscouter* and *Pleader* exert opposite effects on the convergence rate and local optima trapping. Thus, *Pscouter* and *Pleader* are mutually restricted such that one cannot be very much larger or smaller than the other.

Properly balancing the *PScouter* and *Pleader* values is crucial for increasing the convergence speed and enhancing the ability to find the global optimal solution. Increasing the convergence speed must not unduly compromise the capability of finding the global optimum, which itself requires a good random search method. In this paper, the appropriate parameter combination is determined in an experiment.

The appropriate range of *PScouter* and *Pleader* was found in a simulation experiment conducted in R2-01, which can be found in the literature [20]. Assuming 85 bees, the other parameters, whose values were suggested in the literature [40], were employed as follows: maximum iterative time (MIT) = 200; transition rate of onlookers (*γ*) = 0.9, cycle limit = 50, *α* = 1, *β* = 4. *PScouter* was varied as 0, 0.1, and 0.3, whereas *Pleader* was varied as 0.25, 0.33, 0.5, 0.75, and 1. The averages and optimal values were obtained from 10 independent computations. The results are listed in Table 2.

**Table 2:** Comparison results for R2-01 with different parameter values.

*Pscouter*	*Pleader*	average	optimal value
0	0.25	1255.3461	1236.14
0	0.33	1258.5632	1239.26
0	0.5	1257.0142	1236.14
0	0.75	1253.7894	1240.78
0	1	1260.1143	1242.75
0.1	0.25	1253.2104	1238.29
0.1	0.33	1252.3357	1232.34
0.1	0.5	1252.2123	1230.04
0.1	0.75	1262.9213	1244.32
0.3	0.25	1259.3995	1240.78
**0.3**	**0.33**	**1250.3614**	**1229.21**
0.3	0.5	1259.3995	1238.29

From Table 2, we find that (*PScouter*, *Pleader*) = (0.3, 0.33) yielded the best performance.

**Fig 7:**
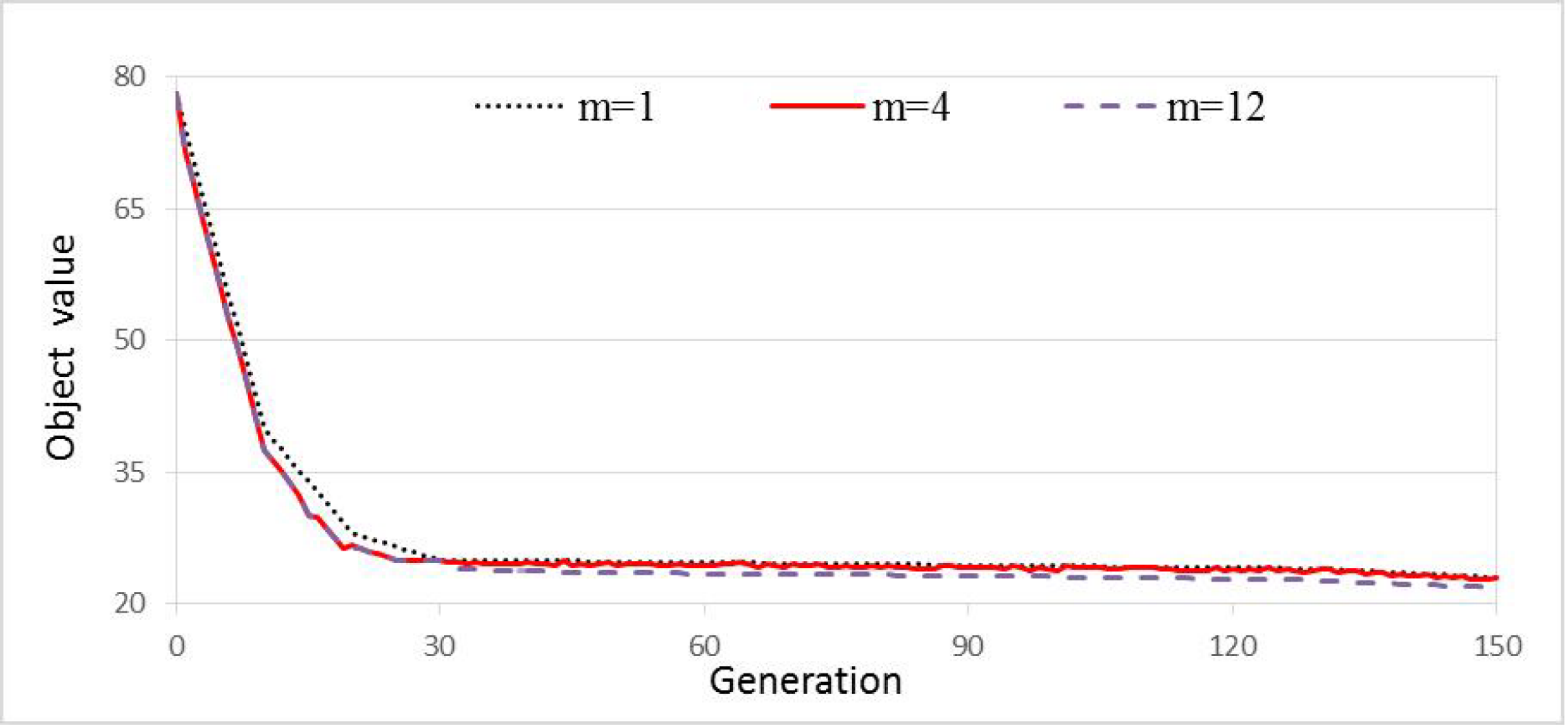
Effect of number of random original food sources *m* on the performance of IABC.

In addition, we randomly created food sources at the initialization step. The number of candidate food sources was increased by a neighborhood moving algorithm. In the experiments, the number of random original food sources *m* affected the convergence speed and accuracy of the algorithm. This simulation experiment was carried out 20 times, setting *m* from 1 to 12, and maintaining the other parameters at their previous values (stated above). And Fig 7 plots the convergence when the problem was solved by the IABC algorithm with *m* = 1,4 and 12. It is concluded that the solution was less accurate at *m* = 1 or *m* = 12 than at *m* = 4. Precise solutions are obtained for *m* ∈ [3,8]. Thus, *m* = 5 was adopted in the remaining experiments.

### Numerical analysis

To check the performance of the proposed IABC algorithm, we employed some well-known benchmarks (Solomon) in the past literatures [9, 40–50]. The results from IABC and the best-known solutions are listed in Table 3. Category C represents clustered problem set. Category R is randomly generated geographical distribution of customer nodes. Category RC is a mix of random and clustered structures. The IABC was comparable to the known optimal solution (bold font in Table 3) in 29 experiments, and surpassed the best-known solutions in 4 experiments (C101, C105, C109, R108). Also, in 10 results, the number of vehicles was clearly lower in IABC than in the other algorithms and the optimal solutions were almost equal, indicating that the IABC lowers the vehicle cost. And the average deviation from best known solutions are 1.64%. The solutions calculated in set C, in which customers are clustered in groups, are encouraging. This may be because the introduction of crossover operation diversifies the bee colony, widens the search space and prevents the algorithm from trapped in local optimization. For most heuristics, it is difficult to find the best solution in local and is easily trapped in local optimal because the similarities between customers in instance set C. The crossover operation with bigger crossover rate can expand solution space and get relatively good solution. Considering both number of vehicles and the total distance, IABC presents as an effective method for solving theoretical and practical VRPTWs.

**Table 3:** Comparison of best-known solution and best IABC.

No.	Reference	Best-known		IABC		
		Number of vehicles	Total distance	Number of vehicles	Total distance	DFB*(%)
C101	Desrochers et al. [42]	10	827.3	**9**	**827.3**	0
C102	Desrochers et al. [42]	10	827.3	**9**	831.5	0.507
C103	Tavares et al. [45]	10	826.3	10	834.4	0.980
C104	Tavares et al. [45]	10	822.9	10	842.1	2.333
C105	Potvin and Bengio [43]	10	828.94	**9**	**828.94**	0
C106	Desrochers et al. [42]	10	827.3	10	833.7	0.773
C107	Desrochers et al. [42]	10	827.3	10	837.2	1.196
C108	Desrochers et al. [42]	10	827.3	10	830.6	0.398
C109	Potvin and Bengio [43]	10	828.94	10	**828.94**	0
R101	Desrochers et al. [42]	18	1607.7	**17**	1618.3	0.659
R102	Desrochers et al. [42]	17	1434	17	1465	2.161
R103	Lau et al. [46]	13	1175.67	13	1207	2.664
R104	Ghoseiri and Ghannadpour [47]	10	974.24	10	996.24	2.258
R105	Rochat and Taillard [43]	14	1377.11	14	1390.5	0.972
R106	Rochat and Taillard [43]	12	1252.03	**11**	1263.12	0.885
R107	Ombuki et al. [48]	11	1100.52	**10**	1126.3	2.342
R108	Tan et al. [40]	10	954.03	**9**	**927.8**	2.749
R109	Chiang and Russell [41]	12	1013.16	12	1028.5	1.514
R110	Rochat and Taillard [44]	11	1080.36	**10**	1088.2	0.725
R111	Ombuki et al. [48]	10	1096.72	10	1099.46	0.249
R112	Rochat and Taillard [44]	10	953.63	10	960.5	0.720
RC201	Thangiah et al. [9]	4	1249	4	1258.6	0.768
RC202	Taillard et al. [49]	4	1164.25	4	1178.9	1.258
RC203	Tan et al. [40]	4	1026.61	3	1083.6	5.551
RC204	Gambardella et al. [50]	3	798.46	3	799.12	0.082
RC205	Tan et al. [40]	4	1300.25	4	1321.3	1.618
RC206	Thangiah et al. [9]	3	1158.81	3	1171.2	1.069
RC207	Ghoseiri and Ghannadpour [47]	4	1040.6	**3**	1096.5	5.371
RC208	Ombuki et al. [48]	4	785.93	**3**	833.97	6.112

* Deviation from the best known solution

The simulation results reveal three main features of the IABC algorithm: 1) the structure of the algorithm is simple and easily understood. It requires minimal preliminary knowledge and has very strong readability. 2) The solution is highly stable. In an unchanging running environment, multiple runs of the algorithm yield the same outcome. 3) The algorithm runs in parallel; that is, every bee simultaneously begins its working cycle from different missions, so the optimal solution is rapidly found in a large solution space. Therefore, IABC is effective for VRPTW problems.

Furthermore, to test the performances of the crossover operation and scanning strategy, we separately introduced these strategies to the standard ABC. The ABCs with the crossover operation and scanning strategy are denoted as ABC-C and ABC-S, respectively. The results are shown in Table 4.

**Table 4:** Computational results using IABC, ABC-C and ABC-S.

No.	ABC-C			ABC-S			IABC		
	Number of vehicles	Total distance	runtime	Number of vehicles	Total distance	runtime	Number of vehicles	Total distance	runtime
C101	9	827.3	69	9	827.3	65	9	827.3	62
C102	9	831.5	68	9	831.5	66	9	831.5	60
C103	10	834.4	80	10	834.4	78	10	834.4	74
C104	10	842.1	118	10	845.6	115	10	842.1	106
C105	9	828.94	52	9	828.94	48	9	828.94	45
C106	10	833.7	75	10	833.7	69	10	833.7	60
C107	10	837.2	116	10	837.2	113	10	837.2	106
C108	10	833.6	70	10	835.3	64	10	**830.6**	58
C109	10	828.94	51	10	828.94	49	10	828.94	45
R101	17	1618.3	375	17	1618.3	368	17	1618.3	341
R102	17	1465	361	17	1465	358	17	1465	341
R103	13	1207	245	13	1207	240	13	1207	229
R104	10	996.24	134	10	996.24	132	10	996.24	122
R105	14	1394.3	325	14	1398.8	325	14	**1390.5**	307
R106	11	1263.12	265	11	1263.12	261	11	1263.12	243
R107	10	1126.3	220	10	1126.3	215	10	1126.3	202
R108	9	972.8	126	9	972.8	123	9	972.8	111
R109	12	1028.5	138	12	1028.5	134	12	1028.5	123
R110	10	1091.5	146	10	1095.9	138	10	**1088.2**	126
R111	10	1099.46	162	10	1099.46	155	10	1099.46	146
R112	10	960.5	123	10	960.5	113	10	960.5	108
RC201	4	1263.1	268	4	1263.1	252	4	**1258.6**	239
RC202	4	1178.9	245	4	1178.9	238	4	1178.9	228
RC203	3	1083.6	146	3	1083.6	136	3	1083.6	125
RC204	3	799.12	35	3	799.12	34	3	799.12	32
RC205	4	1330.7	330	4	1330.7	316	4	**1321.3**	304
RC206	3	1171.2	224	3	1171.2	216	3	1171.2	203
RC207	3	1096.5	164	3	1096.5	159	3	1096.5	146
RC208	3	833.97	74	3	833.97	74	3	833.97	73

Table 4 shows that the ABC-C performs very similarly to IABC, whereas ABC-S obtains the best- known solution in some instances. The quality of the total distance and number of vehicles was comparable in IABC and in ABC-C and ABC-S. However, some of the total distances are better in IABC than in ABC-C and ABC-S, reflecting that the crossover operation and scanning strategy collectively prevent the algorithm from trapping in local optima. The crossover operation improves the solutions, but also increases the computation time. In Table 4, ABC-C consumes more runtime than ABC-S, possibly because the crossover operation requires time to search the crossover nodes. Furthermore, IABC integrates the crossover operation and scanning strategy to provide high-quality solutions in less time than ABC-C and ABC-S.

**Fig 8:**
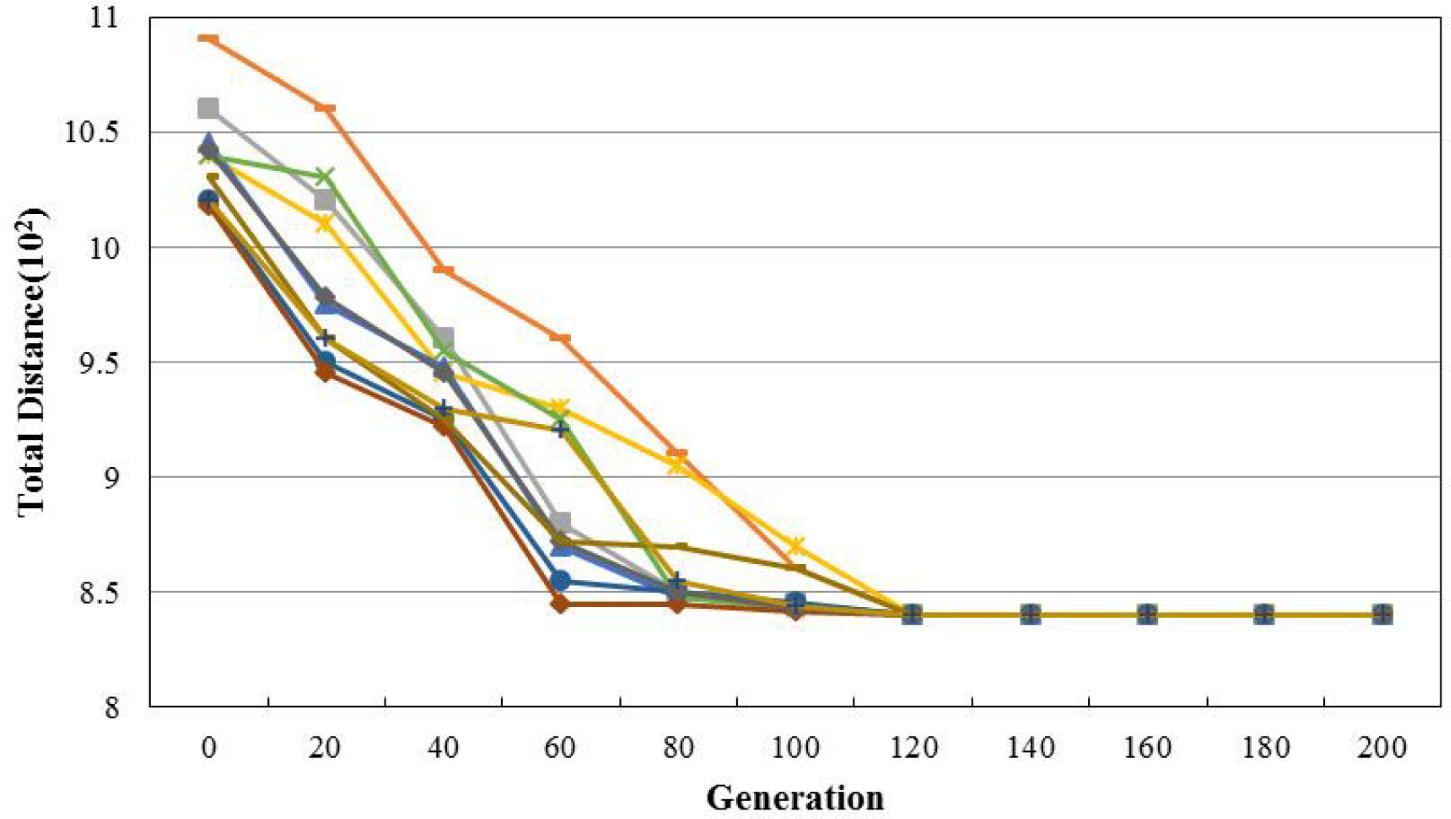
The convergences of IABC.

To analyze the convergences of IABC, the data of C108 is computed by our proposed IABC in ten times and the results can be seen in Fig 8. From Fig 8, it can be attained that the total distance decreases fast before the 60th generation, and then it changes smoothly. The least prediction error appears at about 120th generation, and then it almost remains unchanged. It can be also found that the calculation results of the ten times are almost equal. This suggests that the proposed IABC algorithm has a good converge and this algorithm is effective for the vehicle routing problem with time window.

## Conclusion

This paper develops an improved ABC algorithm for solving VRPTW, a complicated combinatorial optimization problem. ABC algorithms, which randomly search for the optimal solution, have been gradually adopted in combinatorial optimization, artificial intelligence and other fields. Other uses of ABCs are robots that search for optimal routes, scheduling and services that seek to transport fresh food in the best order of delivery. The positive feedback and coordination of the ABC algorithm are effective for distribution systems. This paper improves the performance of standard ABC by a crossover operation borrowed from the genetic algorithm, and a scanning strategy. The Comparison with best-known solution in classic VRPTW experiment verifies the capability of the IABC algorithm. The comparison of the results of ABC-C, ABC-S, and IABC shows that the incorporation of crossover operation and scanning strategy can improve the performance of ABC for VRPTW. However, the crossover operation may increase the computation time for the convergence value with the reason that it requires time to search the crossover nodes. In addition, good performance of IABC for small instances cannot ensure the validity in large instances. In future work, we will improve the convergence speed and demonstrate the effectiveness of the algorithm in large instances and practical case studies. And the performance of the IABC in artificial intelligence and other fields are also expected. Besides, information and communication technology advances have encouraged the development of advanced traveler information systems (ATIS) [51]. Applying ATIS to this paper should be studied in the future.

## Supporting information

S1 Table List of notations. (DOC)
