# Supplementary material for: Improved artificial bee colony algorithm for vehicle routing problem with time windows
Source: PLoS One. 2017 Sep 29;12(9):e0181275. doi: 10.1371/journal.pone.0181275 (PMC5621664; doi:10.1371/journal.pone.0181275)
Supplement: S1 Table — (DOC) [file pone.0181275.s001.doc]

**S1 Table** List of Notations

| *S* | The number of solutions |
| --- | --- |
| *u_i_* | The position of food source *i* |
| $u_{id}$ | The *D*-dimensional coordinate vector of *u_i_* |
| *C* | The cycle number |
| *M* | The maximum number of cycle |
| *S_ij_*(*t*) | The control information for scout |
| *f_ij_*(*t*) | The control information for follower |
|  | The probability that bee *k* travels from node *i* to *j* |
| *l* | The number of unvisited nodes |
| *r* | The guiding strength of the leader |
| *p_m_* | The crossover rate |
| $p_{m}^{min}$ | The minimum crossover rate |
| $p_{m}^{max}$ | The maximum crossover rate |
| *c_i_* | The identifier of client |
| *H* | The number of clients |
| *N* | The number of distribution centers |
| *T* | The maximum iteration generations |
| *t* | The current iteration generations |
| *θ* | The slope of path |
| *z* | The abscissa of the intersection |
| *b* | The judgment parameter |
